# Enrichment and single-cell analysis of circulating tumor cells

**DOI:** 10.1039/c6sc04671a

**Published:** 2016-12-07

**Authors:** Yanling Song, Tian Tian, Yuanzhi Shi, Wenli Liu, Yuan Zou, Tahereh Khajvand, Sili Wang, Zhi Zhu, Chaoyong Yang

**Affiliations:** a MOE Key Laboratory of Spectrochemical Analysis & Instrumentation , The Key Laboratory of Chemical Biology of Fujian Province , State Key Laboratory of Physical Chemistry of Solid Surfaces , Collaborative Innovation Center of Chemistry for Energy Materials , Department of Chemical Engineering , Department of Chemical Biology , College of Chemistry and Chemical Engineering , Xiamen University , Xiamen 361005 , China . Email: cyyang@xmu.edu.cn; b Department of Hematology , The First Affiliated Hospital of Xiamen University , Xiamen 361005 , China; c College of Biological Science and Engineering , Fuzhou University , Fuzhou 350116 , P. R. China

## Abstract

Up to 90% of cancer-related deaths are caused by metastatic cancer. Circulating tumor cells (CTCs), a type of cancer cell that spreads through the blood after detaching from a solid tumor, are essential for the establishment of distant metastasis for a given cancer. As a new type of liquid biopsy, analysis of CTCs offers the possibility to avoid invasive tissue biopsy procedures with practical implications for diagnostics. The fundamental challenges of analyzing and profiling CTCs are the extremely low abundances of CTCs in the blood and the intrinsic heterogeneity of CTCs. Various technologies have been proposed for the enrichment and single-cell analysis of CTCs. This review aims to provide in-depth insights into CTC analysis, including various techniques for isolation of CTCs with capture methods based on physical and biochemical principles, and single-cell analysis of CTCs at the genomic, proteomic and phenotypic level, as well as current developmental trends and promising research directions.

## Abstract 2

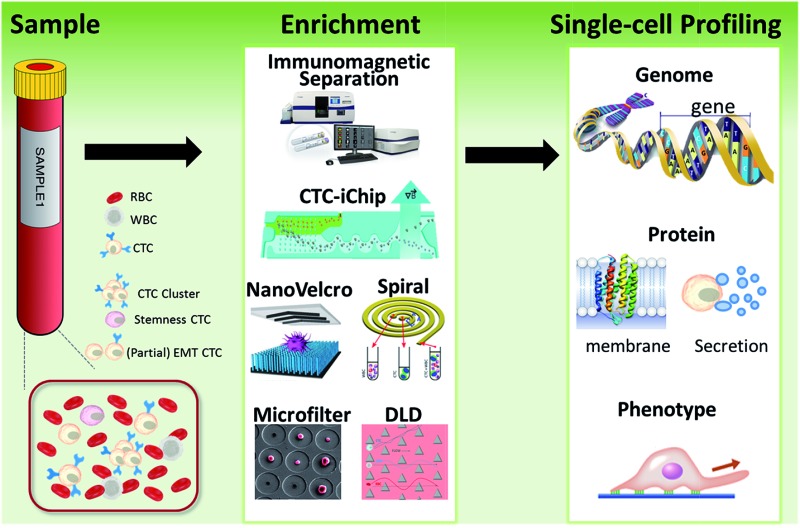
This review aims to provide in-depth insights into CTC analysis, including various techniques for isolation of CTCs and single-cell analysis of CTCs, as well as current developmental trends and promising research directions.

## 1 Introduction

The leading cause of cancer-related mortality is tumor metastasis. Circulating tumor cells (CTCs), defined as the small number of tumor cells spreading through the blood after detaching from the primary tumor, are considered to be responsible for the establishment of distant metastasis for a given cancer.^1–3^ Numerous recent studies have indicated that CTCs may act as a real-time biomarker to better understand disease progression and therapy assessment, complementary to traditional biopsy sampling.^4,5^ As a new type of liquid biopsy, CTC analysis offers the possibility to avoid invasive tissue biopsy with practical implications for cancer diagnostics.

Despite this great potential, until now CTC analysis has barely entered the clinical arena, largely because of the daunting technical challenge in isolating these rare cancer cells with ultrahigh sensitivity and selectivity. The main technical challenge lies in the fact that CTCs are very rare in the bloodstream, with concentrations generally estimated to be several CTCs among billions of red blood cells (RBCs) and millions of leukocytes per milliliter of whole blood.^6^ Thus, highly efficient and selective capture is the first critical step in CTC-based analysis.

**Fig. 1:**
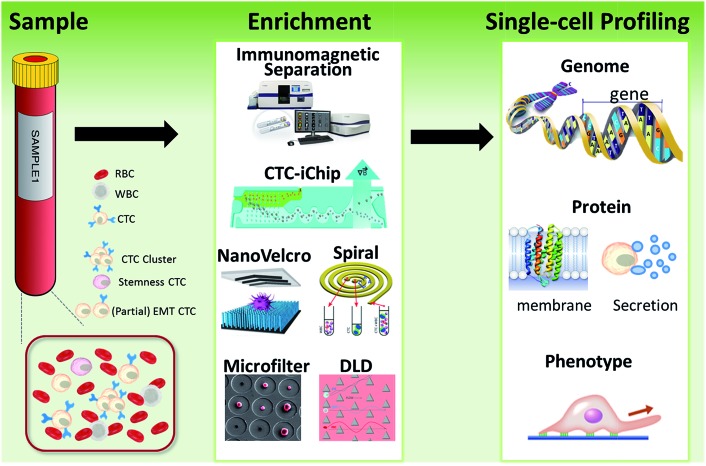
Schematic of current methods for enrichment and single-cell analysis of CTCs. Modified with permission (adapted from ref. 21, 27, 51 and 70, and modified from ref. 25).

Another challenge is the heterogeneity observed extensively in cancer cells.^7^ For example, individual blood cancers exhibit significant intra-clonal heterogeneity, indicating the need for single cell analysis.^8^ Like most tumor tissues, CTCs show distinct morphological and phenotypic features, including potential morphological, genetic, metabolomic, proteomic and metastatic variations (Fig. 1).^9,10^ For instance, to intrude blood vessels, some of the cancer cells may undergo an epithelial to mesenchymal transition (EMT), resulting in progressive loss of the expression of epithelial markers.^11^ This heterogeneity poses the greatest challenge for enrichment, as there is no unique common biomarker for identification. More importantly, heterogeneity of CTCs highlights the importance of analyzing CTCs at the single-cell level, because bulk analysis may lose important information on individual CTCs.^12,13^ Thus, beyond CTC enumeration, the profiling of the phenotype and genotype of single CTCs may provide deeper insights into CTCs, which are important for the identification, origin, evolution and elucidation of cancer metastasis.

This perspective provides a broad picture of CTC analysis, including advanced techniques for enrichment and single-cell analysis of CTCs, as well as current developmental trends and promising research directions (Fig. 1). To date, various techniques have led to exciting opportunities for CTC study. We first summarize the significant progress in CTC enrichment with satisfactory efficiency and purity, with special attention to emerging methods based on microfluidic technologies. We also discuss a number of key platforms for single-cell CTC characterization at the molecular level, including genomic, proteomic and phenotypic profiling and drug screening, which will lead to a comprehensive understanding of CTCs. Finally potential promising research directions regarding CTCs are also discussed.

## 2 Methods for CTC enrichment

The key technical challenge in CTC research is isolation and detection. Detecting a few CTCs among the vast numbers of other cells and differentiating the CTCs from epithelial non-tumor cells and leukocytes represent daunting technical challenges. Many CTC detection platforms utilize physical and morphological features of cancer cells, such as size, deformability, electrical charge or density.^14,15^ In addition, numerous strategies based on specific biological properties, for instance, tumor specific markers, have also been developed for CTC isolation.^13,16^

### 2.1 Traditional methods

Immunomagnetic separation has been the most widely used approach to isolate CTCs from patient samples. Among all the platforms, the CellSearch® system is the first and only clinically validated and FDA (US Food and Drug Administration) cleared test for capturing and enumerating CTCs of metastatic breast, prostate or colorectal cancer. CellSearch® is intended for the enumeration of CTCs of epithelial origin and uses anti-EpCAM-coated magnetic beads. After capture, the enriched cells are treated with a nuclear stain (DAPI^+^), fluorescent antibody conjugates against epithelial markers (EpCAM^+^) and cytokeratins (CK^+^), and a leukocyte marker (CD45^–^) for distinguishing CTCs from white blood cells. CellSearch® has been widely used as an objective indicator of CTC counting for evaluating other new approaches. However, the standard immunomagnetic separation methods based on the recognition of epithelial markers are not usually able to isolate the subpopulation of CTCs undergoing the EMT process, because of their lower or absent epithelial marker expression.^17^ Consequently, potentially more aggressive metastatic cells may actually be less likely to be captured using this principle.^18^

### 2.2 Microfluidic tools

By virtue of its small physical dimensions, enhanced capture efficiency, improved cell viability, and detailed molecular and phenotypical characterization, microfluidic technology has served as a promising platform for the enrichment of CTCs. Recently, researchers have explored many approaches to enrich or separate cells using microfluidic devices based on size, electrophoretic mobility or surface markers of cells.

#### 2.2.1 Physical principles

Physical principles for CTC separation are mainly dependent on differences in physical properties, including size, density, electrical polarizability, or phenotype, between CTCs and leukocytes. These methods can avoid the epithelial antigen bias of immunocapture methods.

##### Microfiltration

**Fig. 2:**
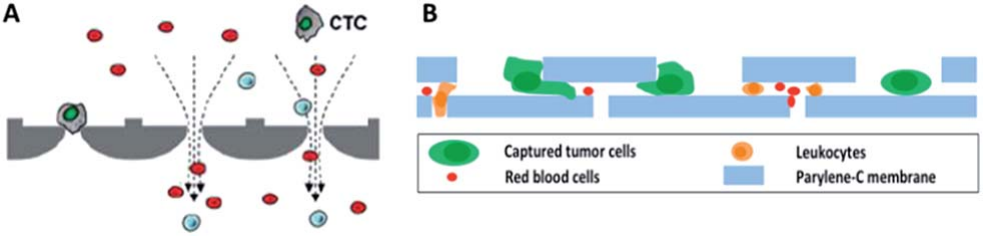
Working principle of microfiltration-based devices for CTC isolation. (A) Size-selective microcavity array (adapted from ref. 21). (B) Separable bilayer microfiltration device (adapted from ref. 23).

Microfiltration, which is based on the assumption that CTCs are larger than leukocytes, has huge potential for achieving high throughput analysis of a large number of samples. CTCs are generally considered to originate from epithelial cells with diameters of 10–20 µm. A pore or slit with a size of around 6 to 10 µm has been proven to be effective for CTC isolation. Zheng and colleagues developed a 2D parylene membrane microfilter device,^19^ achieving 89% CTC identification of 57 cancer patient samples with only 46% identification by CellSearch®.^20^ In another study, Hosokawa *et al.* reported a series of miniaturized devices based on a circular or rectangular microcavity array for highly efficient enrichment of CTCs (Fig. 2A).^21^ Their microcavity array can also be integrated for further analysis, such as on-chip FISH and immunostaining. However, the concentrated tension stress and mechanical trauma at the pore edges of the 2D microfilter device could influence the viability and integrity of cells, making them incompatible for further biological analysis. To prevent cell damage, Zheng *et al.* developed a double-layered 3D microfilter,^22^ in which the bottom pores are shifted from the top pores, resulting in direct support of the trapped cells by the bottom layer to reduce stress concentration on the cell plasma membrane. They also designed a new kind of separable bilayer (SB) microfilter with a special architecture of pore alignment and a specific gap between the two layers (Fig. 2B).^23^ The pores on the top layer are 40 µm in diameter, avoiding entrapment of CTCs inside the top pores. Instead, the CTCs are captured in the gaps at the edges of the large top pores and the bottom layer. This intelligent design can reduce the mechanical stress on captured cells and give greater freedom for migration, proliferation and functional characterization of the captured cells.

##### Hydrodynamics

**Fig. 3:**
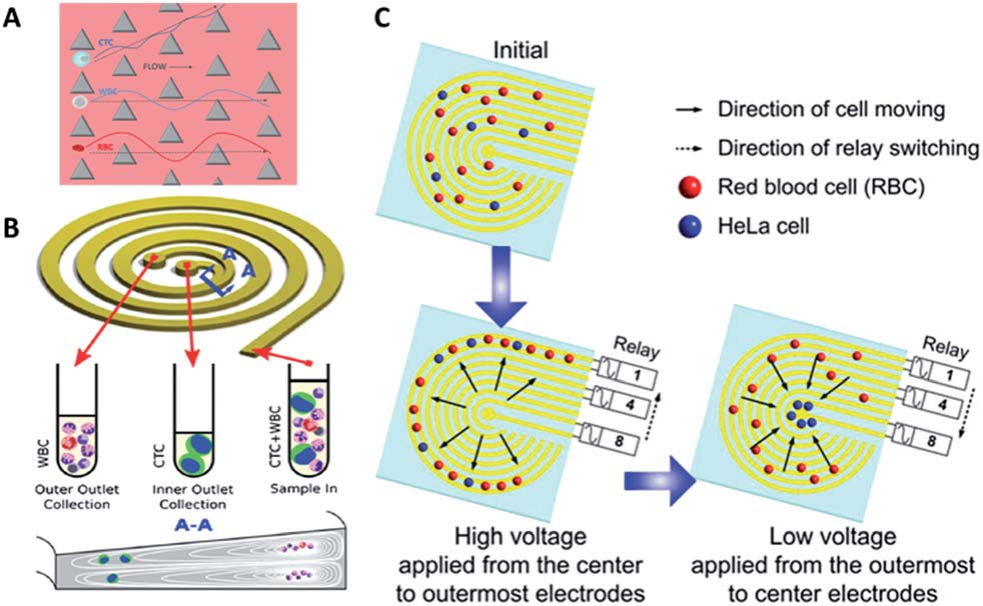
Device design and working principle of hydrodynamics (A and B) and dielectrophoresis (C) for CTC enrichment. (A) DLD array (modified from ref. 25). (B) Spiral channel with a trapezoidal cross-section (adapted from ref. 27). (C) Dielectrophoresis in a stepping electric field (adapted from ref. 35).

Hydrodynamic-based separation of CTCs involves laminar flow in microchannels, in which particles in the fluid move in straight lines parallel to the tube walls. Deterministic lateral displacement (DLD), a type of hydrodynamic-based separation first proposed by Huang *et al.*,^24^ is frequently used for high-resolution separation of particles in continuous flow based on size. In microfabricated array devices, particles travel in two types of paths distinguished by a critical diameter (*D*_c_). Particles larger than *D*_c_ collide with the array of microfabricated posts and shift to the adjacent streamline, while small particles stay within the original streamline (Fig. 3A). Because CTCs are on average larger than leukocytes, DLD is an excellent way to isolate CTCs. In 2012, Loutherback *et al.* designed a DLD chip with a 10 mL min^–1^ flow rate for isolating 10^6^ cancer cells per mL from blood with an efficiency greater than 85%.^25^ Furthermore, Liu and his colleagues integrated DLD parallel multichannels with antibody-based immunocapture to achieve a 1500-fold enrichment at a rate of 9.6 mL min^–1^.^26^ The results indicated that a combination of immunocapture and cell size differentiation is better than cell size differentiation alone for practical CTC separation.

Besides DLD, other kinds of hydrodynamics-based CTC separation have also been reported. A spiral microfluidic device is an emerging tool to separate CTCs from peripheral blood. Warkiani *et al.* reported a slanted spiral for CTC isolation (Fig. 3B),^27^ and successfully isolated spiked cancer cells with more than 80% efficiency in a 7.5 mL sample within 8 min. Kim *et al.* performed a parametric study and numerical simulation of inertial focusing effects and utilized the results to separate CTCs from white blood cells (WBCs) with high purity.^28^ Tanaka and his colleagues designed a multistage microfluidic device to create inertial force instead of a spiral device. The collection efficiency of CTCs was about 85% with 120-fold enrichment.^32^ To achieve a better separation, some researchers combined different separation principles. Bhagat *et al.* combined pinched flow with shear-modulated inertial microfluidics for CTC capture, with more than 80% cell recovery.^29^ Also, a similar study by Jiang's group combined inertial microfluidics with a membrane filter for label-free and size-based enrichment of lung cancer cells.^30^

##### Dielectrophoresis

For contactless and harmless control of cell motion in suspension, dielectrophoresis is the method of choice, in which directional manipulation of polarizable particles is based on their distinct dielectric properties. The significant difference in dielectric properties between CTCs and blood cells offers a platform for CTC isolation. In 1995, Beckerand his colleagues separated MDA-MB-231 breast cancer cells from normal blood.^31^ Gascoyne *et al.* applied dielectrophoretic field-flow fractionation to capture three types of cultured tumor cells spiked in peripheral blood.^32^

However, traditional dielectrophoresis devices are limited by large electrodes, which are not suitable for most microfluidic devices with small physical dimensions. With the development of microfabrication technology, microelectrode patterns have been prepared for electrophoresis.^33,34^ Jen *et al.* designed a curvy electrode pattern to enrich CTCs by forming stepping electric fields, leading to the possibility of using a dielectrophoresis-based microfluidic chip to enrich CTCs in an efficient and flexible manner.^34^ Subsequently, Jen's group utilized circular microelectrodes to generate a stepping electric field (Fig. 3C).^35^ The dielectrophoretic force on HeLa cells is 7-fold larger than that on red blood cells, allowing HeLa cells to be enriched onto the central microelectrode and captured from blood. Alshareef *et al.* reported separation of a mixture of MCF-7 and HCT-116 cells by a microfluidic dielectrophoresis sorter with optically transparent electrodes,^36^ demonstrating that even different types of CTCs could be distinguished based on their dielectric properties. More practically, Fabbri and his co-workers fabricated a dielectrophoresis array in a microchannel to isolate pure CTCs.^37^ They utilized the device to analyze peripheral blood samples from metastatic colon cancer patients with 100% pure cell recovery. To isolate CTCs with higher purity and recovery rate, the dielectrophoresis method was also combined with other separation mechanisms, such as immunocapture^38^ or multi-orifice flow fractionation (MOFF).^39^

Most of the above examples are based on just one physical character difference between cancer cells and blood cells. In addition, some methods that rely on the combination of several physical properties have also been developed. Li *et al.* demonstrated an acoustic-based microfluidic device that was able to isolate CTCs from patient samples, due to the differences in size, density and compressibility causing different amplitudes of the primary acoustic radiation force.^40^

In general, physical CTC separation methods are label-free and high-throughput, and avoid the bias of surface markers. Among them, hydrodynamics has the highest throughput, followed by microfiltration and dielectrophoresis.^15^ The enrichment yields are similar for these three methods.

#### 2.2.2 Immunocapture of CTCs

CTC isolation based on physical principles usually achieves high-throughput without bias regarding the properties of membrane markers, but the purity problem cannot be overlooked. Immunocapture of CTCs takes advantage of the highly specific interaction between capture ligands and antigens specifically present on the surface of CTCs. In most approaches, positive binding ligands are immobilized in microchannels, or on pillars or other nanostructures with enhanced surface-to-volume ratio to achieve high capture efficiency and purity. Most immunoaffinity methods make use of the EpCAM protein for immune interaction, which is a transmembrane glycoprotein overexpressed in most solid cancers, such as liver, breast, pancreatic, stomach, prostate, and colon cancers.^41^ Alternatively, some methods use a tissue specific membrane protein, for example, prostate-specific membrane antigen (PSMA) for prostate carcinoma or epidermal growth factor receptor 2 (HER2) for breast cancer.

##### Antibodies

**Fig. 4:**
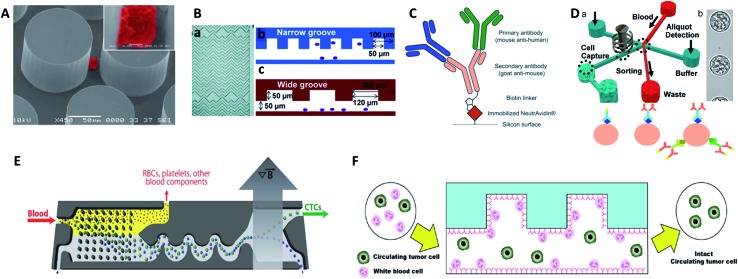
Device design and working principle of antibody-based immunocapture for CTC enrichment. (A) CTC-chip (adapted from ref. 42). (B) HB-chip (adapted from ref. 44). (C) Silicon surface modified with primary antibodies and a secondary antibody linker (adapted from ref. 47); (D) eDAR modified with an antibody cocktail (adapted from ref. 48 and 49); (E) CTC-iChip (adapted from ref. 51); (F) GASI-chip (adapted from ref. 50).

Antibodies are the most common molecular recognition ligands. The combination of antibodies and microfluidics has been demonstrated as an effective strategy for CTC capture. Toner's group designed a unique microfluidic platform (CTC-chip) capable of effective capture of viable CTCs with high purity in a single step from non-pretreated blood samples (Fig. 4A).^42^ The CTC-chip contained an array of more than 70 000 anti-EpCAM coated microposts to enhance the cell–antibody interaction, resulting in successful identification of CTCs with 99% efficiency from different kinds of cancer samples. However, the large number and complex structure of the microposts posed a challenge for large-scale production and clinical research. Therefore, Toner's group further developed a herringbone-chip (HB-chip) that did not require complex micropost geometry.^43^ The high-throughput microvortex HB-chip relied on transverse flows within microchannels to realize maximized collisions between cancer cells and the antibody-coated chip surface. The low shear design of the HB-chip successfully isolated CTC clusters from a subset of prostate cancer specimens. Furthermore, Sheng and co-workers optimized herringbone mixers by increasing the groove width to reduce trapping of non-target cells (Fig. 4B).^44^

Gleghorn and colleagues developed the geometrically enhanced differential immunocapture (GEDI) technique, which takes advantage of size-dependent collision frequency and antibody-coated 3D posts.^45^ Using GEDI, smaller blood cells are displaced onto streamlines while larger tumor cells are impinged by the posts multiple times, achieving high capture efficiency and purity. Since simply isolating and counting CTCs is insufficient for further studies on the heterogeneity of CTCs, Kelley *et al.*developed a new device that allowed the trapping of CTC subpopulations based on different numbers of magnetic nanoparticles labelled on cells, with the amount depending on the expression level of the surface EpCAM target.^46^ By controlling the linear velocity, high EpCAM-expressing cells with numerous magnetic nanoparticles were captured in the high linear velocity region, while low EpCAM-expression cells with fewer magnetic nanoparticles were captured in the low linear velocity area. By spatially sorting the discrete CTC subpopulations, this approach shed light on the study of EMT in patient CTCs, with higher sensitivity than the gold standard CellSearch® system.

For more comprehensive CTC enrichment, much attention has been focused on the choice of antibodies. By combining the anti-EpCAM and anti-MUC1 (mucin 1) antibodies in a single GEDI microdevice, effective capture of circulating pancreatic cells and pancreatic circulating tumor cells was achieved with a higher efficiency than that achieved by single marker immunocapture (Fig. 4C).^47^ Johnson *et al.* also demonstrated the capture of high and low antigen-expressing breast cancer cells based on an antibody cocktail scheme.^48^ The cocktail of CTC-specific primary antibodies contained antibodies against the epithelial markers EpCAM and EGFR, breast cancer antigen HER2, and mesenchymal marker N-cadherin. By virtue of the combination of the cocktail labelling scheme and high-throughput (3 mL h^–1^) in the ensemble-decision aliquot ranking (eDAR) technique,^49^ the approach reliably demonstrated 6-fold increased recovery of breast CTCs compared to single-step EpCAM staining (Fig. 4D).

Although most research has focused on positive selection for CTC isolation, positive isolation suffers from several limitations, such as lack of heterogeneous information on the target cells and a decrease of cell viability during the detachment process. In order to overcome these problems, Jung's group proposed a negative capture chip that was designed using a geometrically activated surface interaction (GASI) with an asymmetric HB-chip to increase the surface interaction between the non-target cells and the anti-CD45 immobilized channel surface (Fig. 4F).^50^ This novel approach enabled capture of CTCs from peripheral blood from a vast variety of sources. In addition, Toner and co-workers combined negative immunomagnetic separation with physical isolation to develop the CTC-iChip.^51^ This chip was composed of two parts, where chip 1 used DLD to separate nucleated cells from whole blood, and chip 2 used internal focusing to line up cells obtained from chip 1, followed by the immunomagnetic separation of bead-labelled WBCs and unlabeled CTCs (Fig. 4E). Combining the merits of positive and negative isolation, they achieved high purification and a 97% yield of CTCs with large sample volumes.

Although antibodies have been used widely as the identifying molecule in immunocapture of CTCs, there are still some problems which cannot be ignored, such as the batch difference, high cost, thermal stability and release process, which pose challenges for promoting CTC detection in clinical applications.

##### Peptides

Compared with antibodies, peptides are small, stable, and easy to synthesize in large amounts. A peptide which recognizes a specific cell surface receptor or biomarker can be easily obtained through widely used techniques, such as phage display, mRNA display and ribosome display. Wang's group identified a CTC recognition peptide by cell-based selection, called Pep10, which can specifically recognize EpCAM with a binding affinity (*K*_d_ 1.98 × 10^–9^ mol L^–1^) comparable to that of EpCAM antibody (*K*_d_ 2.69 × 10^–10^ mol L^–1^).^52^ By attaching Pep10 to magnetic nanoparticles (MNPs) *via* biotin–avidin interactions, Wang's group demonstrated that Pep10@MNPs (200 nm) had high capture efficiency (above 90% with a purity of 93%) for breast, prostate and liver cancer cells from spiked blood samples.

Peptides are promising identifying molecules for CTC detection. Although peptides have been applied in cancer targeting and as drug molecules, as well as gene carrier vehicles, the clinical use of peptides still faces some challenges. For example, their conformational flexibility and small structures sometimes lead to reversible and weak interactions with limited selectivity.

##### Aptamers

Recently, advances in combinatorial chemistry have enabled widespread research into chemical antibodies known as aptamers, single stranded DNA/RNA molecules that can specifically bind to targets of many types (small molecules and ions, macromolecules, and even whole cells).^53^ Aptamers exhibit many superior qualities, including high thermal stability, small size, high affinity, low cost, simple modification, little to no batch variation, and release mechanisms without damage, making them superior to antibodies as identifying molecules for CTC enrichment. Aptamers for CTC identification can be evolved against cell membrane targets, such as those against EpCAM,^54^ EGFR,^55,56^ and PSMA.^57^ Aptamers can also be selected against whole cancer cells without prior knowledge of the target molecule on the cell surface.^58,59^

**Fig. 5:**
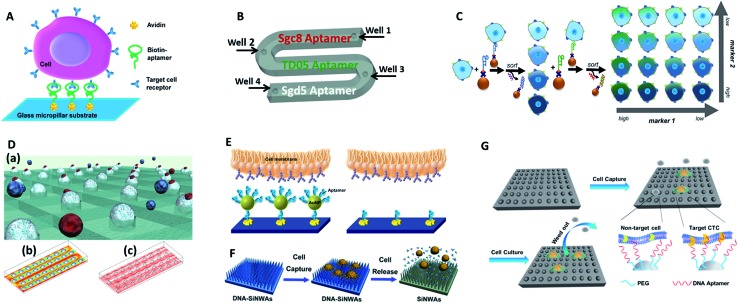
Device designs and working principles of aptamer-based immunocapture (A–D) or 3D surfaces (E–G) for CTC enrichment. (A) DNA aptamer-modified micropillar-based microfluidic device (adapted from ref. 60). (B) Multiplexed detection microfluidic device (adapted from ref. 58). (C) Aptamer-mediated 2D sorting chip (adapted from ref. 62). (D) Hele-Shaw microfluidic chip with anti-EGFR aptamer-modified glass beads (adapted from ref. 61). (E) AuNP aptamer-modified surface (adapted from ref. 65). (F) Aptamer-modified silicon nanowire arrays (SiNWAs) (adapted from ref. 68). (G) Chitosan nanoparticle substrate (adapted from ref. 74).

Tan's group reported an aptamer-functionalized micropillar array in accordance with DLD-based particle separation to enhance the probability of target cell–micropillar interactions.^60^ The biotinylated aptamers were modified onto avidin-adsorbed channels *via* the biotin–avidin interaction (Fig. 5A). This device isolated as few as 10 tumor cells from 1 mL of whole blood, yielding greater than 95% capture efficiency with 81% purity within 28 min. Additionally, about 93% of the isolated cells were viable, meeting the requirement for further molecular and genetic analysis. To further demonstrate the practicality of aptamer functionalized devices, Tan's group extended their previous design to provide multiplexed detection (Fig. 5B).^58^This method had the capability to simultaneously capture multiple cell types, distinguishing three different target cells into their corresponding regions. Similarly, Wan and colleagues designed a Hele-Shaw microfluidic chip with anti-EGFR aptamer-modified glass beads (GBs) (Fig. 5D).^61^ The curved hemispherical surfaces altered the flow in the channel by changing the shear stress around the GBs. The special geometry allowed the linear reduction of fluidic shear stress along the longitudinal axis. Consequently, various regions with varying overall shear stress helped cancer cells with different levels of EGFR over-expression find the right sweet spots, providing multiple levels of binding opportunities.

One of the most attractive benefits of employing aptamers is that the cancer cells captured by aptamer probes can be released gently using nucleases or the corresponding antisense oligonucleotides, thereby effectively maintaining cell viability for downstream analysis. For example, an aptamer and antisense-mediated method that provided two-dimensional (2D) isolation of cancer cell subpopulations was reported by Kelley's group (Fig. 5C).^62^ Cells were first identified by MNPs modified with an EpCAM aptamer to sort the isolated cells into four subpopulations according to their level of epithelial marker expression. The four subpopulations were then released using a complementary oligonucleotide and subsequently tagged with HER2 aptamer functionalized MNPs to divide them into 16 subpopulations. Using this antisense-triggered release, this new technique can be used to separate phenotypic subsets of CTCs according to the expression level of two different markers in a 2D format.

Overall, CTC isolation using aptamers shows enormous potential, but several issues limit translation to clinical applications, including the diversity and stability of aptamers. Future work will involve the selection of a wide variety of aptamers against target CTC biomarkers, cancer cells or marker-specific subtypes of CTCs. On the other hand, stable probes in whole blood are in urgent need for the improvement of the present methods.

##### 3-Dimensional surfaces

The ongoing development of nanotechnology provides exciting opportunities for improving CTC enrichment in microfluidic devices. 3D nano-surfaces offer the opportunity for assembling multiple ligands and providing larger surface areas that substantially enhance the capture efficacy of target cells. In one attempt, Hong's group reported a strategy using nanoscale poly(amidoamine) (PAMAM) dendrimers and anti-EpCAM to significantly increase binding ability.^63,64^ In a parallel-plate flow chamber, the surface with dendrimers significantly enhanced capture efficiency (1.7–3.7-fold compared to epoxy-functionalized surfaces) for different kinds of breast cancer cells. Alternatively, Tan's group chose gold nanoparticles (AuNPs) as efficient high-affinity vehicles to assemble multiple aptamers (DNA nanospheres) (Fig. 5E).^65^ The easy synthesis of AuNPs and mature DNA conjugation methodology enabled up to 95 aptamers to be attached to each AuNP. With a laminar flow flat channel microfluidic chip, the capture efficiency of spiked cell mixtures increased from 49% (by aptamer alone) to 92% (by aptamer AuNPs). Furthermore, aptamers can be easily amplified by enzymatic reaction. Zhao and coworkers engineered a platform with 3D DNA networks containing repeating aptamer units by rolling circle amplification.^66^ The 3D aptamer networks bound to target cells in a cooperative manner to increase affinity, and the special 3D arrangement enhanced the possibility of interactions to allow cell isolation under high flow rates.

Biomimetic nanostructured substrates direct the local topographic interactions between the substrates and nanoscale cellular surface components (microvilli, filopodia), offering a compatible environment for cancer cell attachment. So far, the following 3D nano-surfaces have been used as ultrasensitive platforms in CTC detection: silicon-nanopillars,^67^ silicon-nanowires (Fig. 5F),^68^ graphene oxide,^69,70^ carbon nanotubes,^71^ and polymer nanofibers.^72^ As one of the representative examples, Tseng's group developed a new CTC-capture platform, which integrated an antibody modified nanostructured silicon substrate with an overlaid chaotic micromixer.^73^ In particular, “soft” materials have better cellular compatibility because they match the soft nature of cells to ensure their maximum viability. In Sun's work, aptamer and PEG-modified chitosan nanoparticles were capable of enriching cancer cells from 1 mL WBC solutions with a subsequent *in situ* culture, which removed the non-target cells while proliferating and purifying rare CTCs (Fig. 5G).^74^

These examples demonstrate the potential of biomimetic nano-surfaces as new tools to alleviate the current CTC isolation bottleneck. Nevertheless, up to now, the preferred nanostructure local topographic interaction mechanism is still unclear. Another limitation is the as-yet uncharacterized morphology of isolated cancer cells on nano-surfaces. In addition, non-specific capture is another obstacle for further clinical trials. More thorough studies are expected in the future.

## 3 Release of captured CTCs

**Fig. 6:**
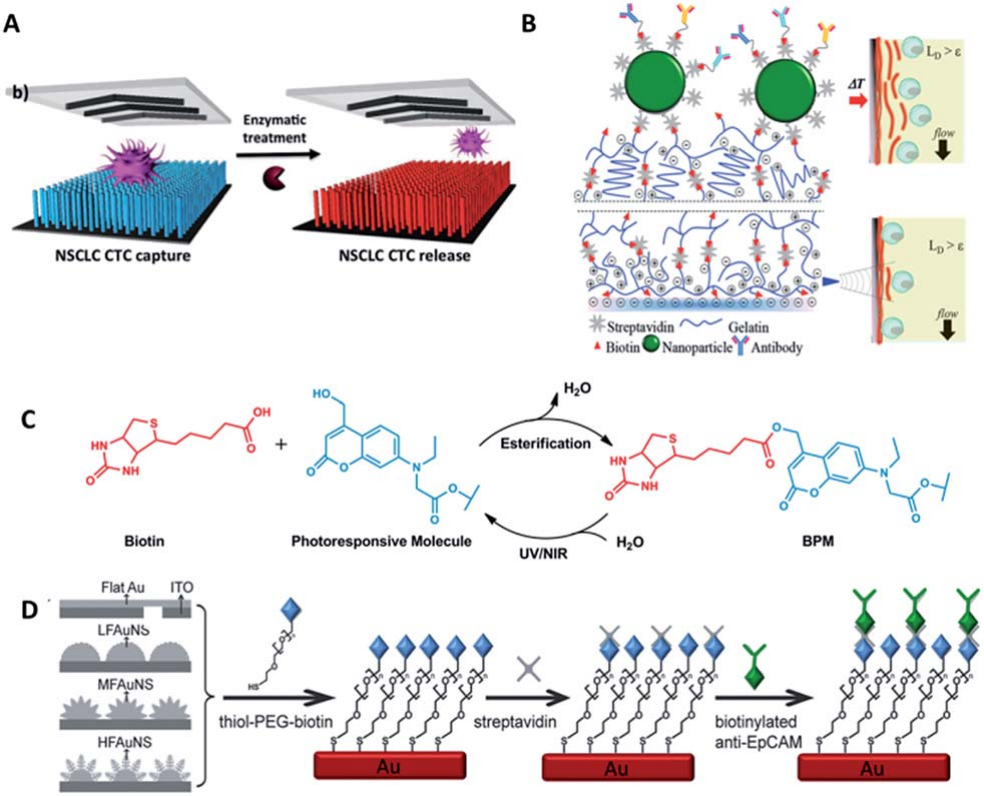
Schematic diagram of release mechanisms. (A) Aptamer-coated NanoVelcro Chip that releases CTCs by enzymatic treatment (adapted from ref. 70). (B) A dual-mode gelatin-based nanostructured coated CTC capture substrate that can achieve temperature-responsive release (top) or mechanosensitive release (down) (modified from ref. 75). (C) Photo-induced cleavage of biotin-photoresponsive molecules (modified from ref. 76). (D) Nanostructured interfaces modified by thiol-PEG-biotin molecules, which can be cleaved by electrical potential (modified from ref. 78).

Most studies so far have emphasized the enumeration of CTCs, while more attention should be paid to the downstream analysis of the cancer cells, such as proliferation, genotyping and drug sensitivity tests. Controlled cell release without damage is significant for subsequent cell analysis. In order to extract useful information from CTCs, release technologies have been developed based on the requirement of high release efficiency and nondestructive recovery of captured CTCs. When using antibodies as capture agents, most CTC recovery methods depend on the harsh proteolytic digestion of the extracellular domains of membrane antigens. The most common reagents are trypsin or EDTA. Unfortunately, this type of release process influences cell viability to some extent. Thus it is necessary to precisely control the reaction time and reagent concentration. In contrast, when aptamers are designed as capture ligands, the release process is gentle and involves changing the secondary structure of aptamers *via* nuclease-degradation or complementary sequence displacement. Iqbal's group applied an EGFR aptamer with a toehold to isolate human glioblastoma cells and achieved 92% release efficiency by a combination of soft shaking and addition of the complementary RNA strand.^61^ The anti-RNA sequence was complementary not only to the aptamer but also to the toehold part, thus ensuring that the recovered cells remained in their native state. Furthermore, by taking advantage of this low-impact release feature, the purity of CTCs can be gradually improved by using two rounds of the cell capture/release process. Shen *et al.* demonstrated that the capture purity can be improved from 15 ± 5% to greater than 95% *via* two rounds of capture/release, and the recovered cancer cells exhibited 78–83% viability (Fig. 6A).^70^

Furthermore, other attempts have been tried. Stott's group developed a dual-mode gelatin-based nanostructured coating formed by layer-by-layer deposition of streptavidin and biotinylated gelatin. Recovery was enabled by temperature change or mechanosensitive response (Fig. 6B).^75^ For bulk-population recovery, increasing the device temperature to 37 °C solubilizes the surface nano-coating as a result of the formation of intermolecular alpha-helical structures between the gelatin molecules and surrounding water. For single-cell release, the localized region can be disrupted by applying mechanical stress through a frequency-controlled micro tip. The newly developed material responds to both thermal and shear stress, making it suitable for different biomedical applications.

Recently, photochemistry has drawn much attention in the field of targeted delivery and photo-switched cell motility. Song *et al.* constructed an antibody-photoresponsive system (Fig. 6C). Under UV and NIR light irradiation, about 73% and 52% release efficiencies were obtained with cell viabilities of 90% and 97%, respectively.^76^ Besides modification of affinity agents, use of a photoresponsive substrate is another choice. Zhao *et al.* combined NanoVelcro Chip and ArcturusXT laser capture microdissection (LCM) technology to capture and recover CTCs. The LCM film meant that 355 nm UV laser cutting could be used to recover the captured cells from the substrate surface for whole exome sequencing.^77^ Electrochemical technology is also applicable to CTC recovery (Fig. 6D). Fractal gold nanostructured (FAuNS) interfaces were functionalized with thiol-PEG-biotin molecules for the introduction of streptavidin, and biotinylated anti-EpCAM was then modified onto the FAuNS surfaces. After CTC capture, 98% release of captured cells with 95% viability could be achieved from the anti-EpCAM coated substrates through electrochemical breaking of the Au–S bonds due to the dissociation of thiol-PEG-biotin molecules from FAuNS interfaces.^78^ This new type of bio-interface can achieve efficient recovery of isolated cancer cells with high viability *via* an electrochemical process and is promising for the development of a new type of bio-nanomaterial.

The above examples have achieved highly efficient CTC release, and the recovered cells exhibit high cell viability. Among the various methods, aptamers may be promising candidates owing to their high capture efficiency, as well as their high release efficiency and nondestructive recovery. However, some drawbacks, such as long processing times and the need for the sophisticated design of aptamer sequences, have limited their application. Innovative release methods still need to be the focus of major research efforts.

## 4 Single CTC profiling

**Fig. 7:**
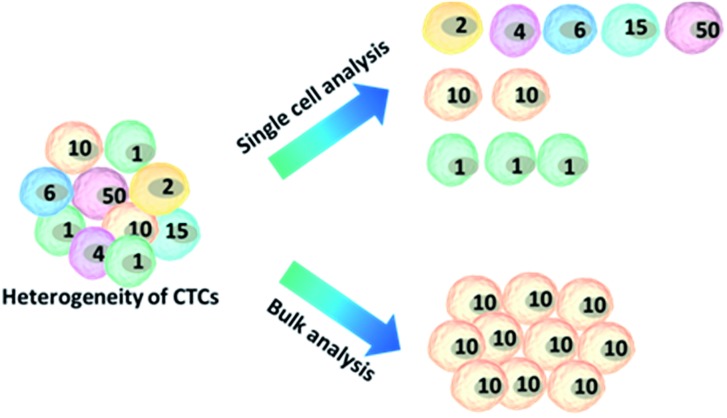
Advantages of single-cell analysis. Comparison of single-cell *versus* bulk analysis results. The number of cells indicates the cellular heterogeneity within a cell population. The bulk analysis shows an average result, losing the real features of the cells, while analysing cell ensembles individually leads to the accurate representation of cell-to-cell variations.

Because of the considerable heterogeneity of CTCs, it is essential to analyze the molecular and genetic properties of single CTCs, because bulk analysis may result in a information loss and misconceptions (Fig. 7).^12,13^ Besides CTC enumeration, the profiling of CTCs at the single-cell level according to phenotype and gene expression can help better understand CTCs, as well as the origin, function, evolution and mechanism of cancer metastasis. However, most of the genetic analysis of CTCs has been performed on nucleic acids extracted from captured CTC populations with limited sensitivity due to leukocyte contamination.^79^ Such bulk analysis cannot determine the degree of heterogeneity across these partially purified cells. For example, in only one out of eight non-small-cell lung cancer (NSCLC) patients who were confirmed to have an EGFR mutation could the mutation be detected in the bulk CTC population.^80^ Single CTC profiling can overcome the restriction proposed by blood cell contamination, enabling the study of CTC heterogeneity.^81^ Recent research has shown a growing trend in CTC capture and single-CTC analysis in sequence or simultaneously. Many single CTC profiling platforms have been established utilizing genomic, proteomic and phenotypic profiling.

### 4.1 Whole genome profiling

Compared with simple enumeration of CTCs, sequencing-based analysis can provide more pertinent information about tumor origin and development. Genomic analysis of CTCs as a noninvasive means of diagnosis provides a valuable aid to clinicians, because it is impractical to undertake repeated biopsies during treatment. The evolution of next-generation sequencing (NGS) technology has promoted the development of genomic and transcriptomic CTC characterization.^82–84^ However, it is difficult to amplify the whole genome at the single-cell level from a very limited number of captured CTCs. To meet this challenge, several whole genome amplification (WGA) techniques have been expanded to raise the amplification uniformity across the entire genome for NGS. To date, multiple displacement amplification (MDA), a WGA method using the Phi29 polymerase, has been widely applied to obtain high physical coverage (>90%) from a single-cell exome or genome.^85–89^ Heitzer *et al.* ^90^ isolated CTCs from colorectal carcinoma patients by CellSearch® and then used MDA to amplify the whole genome for comparative genomic hybridization array analysis and next-generation sequencing. They found that some important driver gene mutations of the primary tumor and metastases were also discovered in the corresponding CTCs. Similarly, mutations originally found in CTCs also existed at the subclonal level in the primary tumors and metastases from the same sample. Nevertheless, MDA generates amplification bias, which causes non-uniform coverage and distortions in reading depth. Another single cell DNA amplification method is multiple annealing- and looping-based amplification cycles (MALBAC) proposed by Xie's group in 2012.^91,92^ This method reduces the amplification bias, making it more suitable for single-nucleotide variations (SNVs) and copy number profiling. Later, they applied MALBAC for genomic analyses of single CTCs from 11 patients with lung cancer. They compared the exome profiles of individual CTCs, and found that SNVs were heterogeneous from cell to cell. In contrast, the copy number variations (CNVs) were specific to cancer types, and individual CTCs from the same patient exhibited reproducible CNV patterns. Based on these findings, they proposed that CNVs at certain genomic loci were selective for metastasis.

**Fig. 8:**
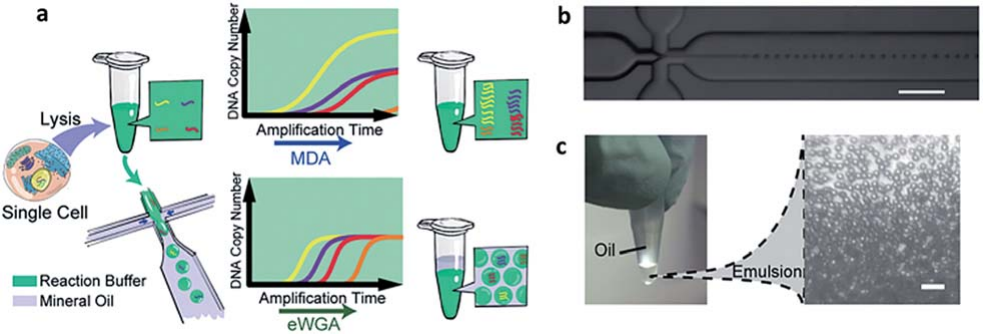
(a) A single cell is lysed and reacted in a tube. The sample is either directly used for conventional MDA, generating amplification bias, or used for eWGA, which occurs in aqueous-in-oil droplets with better amplification uniformity. (b) Aqueous-in-oil droplet generation. Scale bar: 300 µm. (c) Aqueous-in-oil droplets are stable during the MDA reaction. Scale bar: 100 µm (adapted from ref. 93).

The application of MALBAC as a WGA technique has proven to be superior to other approaches. However, the genome coverage and anti-interference of pollutants need to be improved further by developing new WGA methods. Based on the limits of MDA and MALBAC, Huang's group reported emulsion whole-genome amplification (eWGA), which enabled amplification of single-cell genomic DNA fragments in numerous picoliter aqueous-in-oil droplets with good uniformity (Fig. 8).^93^ In addition to suppressing the amplification bias, this approach also offered high coverage of the whole genome and enabled accurate simultaneous detection of CNVs and SNVs. Besides the development of WGA techniques, the advance of more efficient and less invasive cell lysis systems is another crucial area that needs further research effort.

### 4.2 RNA profiling

In addition to whole genome sequencing, single-cell transcriptome sequencing methods have led to significant advances over the past 5 years. In 2012, Sandberg's group established a robust mRNA-Seq method (Smart-Seq) for full-length transcriptome analysis, which has improved the transcriptome coverage.^94^ They applied this method for melanoma CTCs and discovered distinct gene expression patterns, including potential biomarkers and the genes associated with avoiding immune surveillance. This study served as a valuable demonstration to highlight the power and utility of RNA-Seq for single CTC cell study. Similar works were also conducted on breast cancer. Powell and colleagues transcriptionally profiled single CTCs enriched by an immunomagnetic isolation device (MagSweeper).^10^ Their results indicated significant heterogeneity of cancer-associated genes among individual CTCs.

**Fig. 9:**
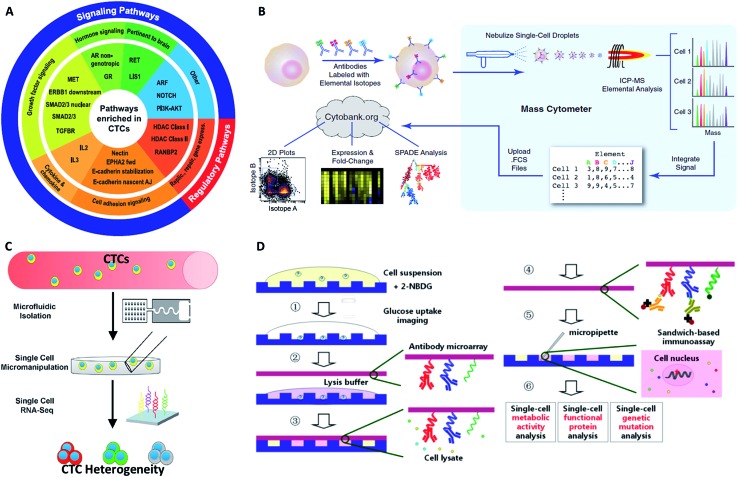
The profiling of CTCs at the single-cell level in terms of gene (A and B) and protein expression (C and D). (A) PID up-regulated molecular pathways enriched in prostate CTCs (adapted from ref. 95). (B) Mass cytometry profiling of immune cell membrane proteins (adapted from ref. 100). (C) Expression of extracellular matrix genes of single CTCs determined by RNA sequencing (adapted from ref. 96). (D) Scheme of single-cell co-detection of glucose uptake, intracellular functional proteins, and genetic mutations using a nanowell array and barcode slide (adapted from ref. 102).

Recently, Maheswaran and Haber conducted single-cell transcriptome profiling of 77 intact prostate CTCs isolated by CTC-iChip and applied the Pathway Interaction Database (PID) to identify key signaling pathways up-regulated in prostate CTCs *versus* primary tumors (Fig. 9A).^95^ They also found expression heterogeneity of the androgen receptor (AR) gene and splicing variants among individual CTCs. Compared to the untreated group, the patients treated with an AR inhibitor showed activation of noncanonical Wnt signaling, which modulated enzalutamide treatment sensitivity. Thus, the striking heterogeneity of AR signaling revealed by single-cell transcriptome sequencing could identify potential clinically related principles of acquired drug resistance. Similar works were also completed by Ting *et al.* for fully defining the composition of pancreatic CTCs (Fig. 9C).^96^ They performed single-cell RNA sequencing of pancreatic CTCs enriched by CTC-iChip, revealing three distinct populations and suggesting multiple routes in the metastatic cascade. Extracellular matrix genes are highly expressed in CTCs, contributing to distal spread of pancreatic cancer.

The above examples have enabled important progress in single-cell RNA profiling, particularly in transcriptome sequencing. However, most of the known single cell RNA sequencing technologies are limited to profiling mRNAs with poly(A) tails, resulting in a certain number of non-polyadenylated RNAs in CTCs being neglected. In particular, many types of microRNA are referred to as “oncomirs”, associated with important cellular functions.^97^ Furthermore, a new kind of non-coding RNA, circular RNA (circRNA)^98^ without poly(A) tails, may play an important role in many biological activities. Therefore, universal transcriptome analysis is urgently needed for all RNA species.

### 4.3 Proteomic profiling

Although proteins are always in higher abundance than nucleic acids, they are more difficult to quantify for CTCs at the single-cell level because of the inefficient molecular amplification of target proteins and the transient nature of certain functional proteins. Nonetheless, as proteins are the final executors of life functions, a comprehensive description of proteins is crucial for fully understanding the behavior and mechanism of CTCs. The classical protein analysis of captured cells is immunofluorescence imaging of surface markers, and this method also enables CTC identification by antibody staining (DAPI^+^/EpCAM^+^/CK^+^/CD45^–^).^99^

Although immunofluorescence imaging is readily available, the simultaneous analysis of multiple protein types is usually limited in terms of quantity. One solution to this issue relies on single-cell “mass cytometry”, which utilizes transition element isotopes as chelated antibody tags for measuring 34 parameters simultaneously in each single cell by atomic mass spectrometric analysis (Fig. 9B).^100^ With the merits of high resolution, little interference from spectral overlap and nebulization into single-cell droplets by the inductively coupled argon plasma, mass cytometry can reasonably be expected to provide system-wide views of protein profiling and precise drug responses at the single-cell level. In another development, Fan's group combined spatial (spots) and spectral (colors) encoding for simultaneous measurement of 42 secreted proteins from single cells, reaching the highest multiplexing record level so far. The subnanoliter microchamber array design guaranteed isolation of individual cells to maintain sufficient protein concentrations.^101^ The antibody barcode array slide contained 15 independent lines, and each line was coated with three types of antibodies with different fluorescent dyes to establish the co-detection of 42 targeted proteins and 3 controls. These single-cell barcode chips were versatile, flexible and information-rich tools to enable different applications, such as immunoassays of membrane, intracellular or secreted proteins. Zhang and his colleagues modified the coating process of the antibody barcode array slide by coating additional poly-l-lysine (PLL) before patterning the antibody barcode (Fig. 9D).^102^ The fresh PLL surface enabled the immobilization of more antibodies on the layer, thereby maximizing the effective limit of low abundance protein detection. They captured CTCs from non-small cell lung cancer peripheral blood using a HB-chip, followed by release of the cells into nanowells using external magnets. As a result, more than 80% of the released CTCs trapped in individual chambers were simultaneously analyzed for 8 intracellular proteins.

Powerful tools such as mass cytometry and single-cell barcode chips have appeared during the past few years, but new approaches for single-cell functional proteomics are still eagerly needed. A major bottleneck involves the degree of multiplexing attainable by current strategies. The current methods reach a maximum number of 45 proteins per cell, which is only a small proportion of the proteome. This restriction is due to the reliance on antibody recognition mechanisms. To break this limit, a new protein detection molecule with high affinity, low cost, and easy accessibility is needed. Moreover, an increased emphasis on sensitive and quantitative analysis methods will clearly push forward the development of ultra-low abundance protein profiling.

### 4.4 Phenotype profiling

While genetic and molecular profiling of CTCs may reveal detailed information about CTC onset and progression, the complete molecular mechanism of cell metastasis is currently still not clear. Therefore, improved techniques for analyzing cellular phenotypes may be effective means to gain insight about CTCs. Comprehensive analysis of the biophysical properties of CTCs and exploration of their influence on cell motility may provide deeper insights into CTCs and metastasis. The strong capacity of cell invasion is thought to have a crucial role in metastasis, as it is related to cell deformability, adhesion and tumor microenvironment.

#### 4.4.1 Motility

**Fig. 10:**
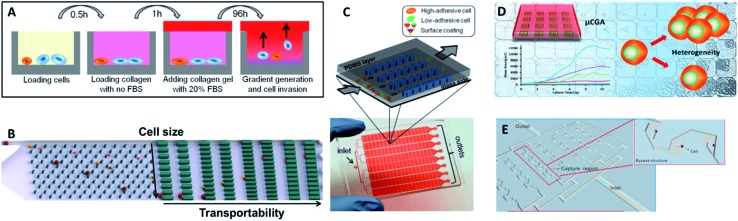
(A) Design of the multiwell invasion chip for 3D cell invasion studies (adapted from ref. 103). (B) Schematic diagram of the biomimetic microfluidic cytometry platform based on cell size and transportability (adapted from ref. 105). (C) Schematic diagram (top) and photograph (bottom) of the high-throughput cell-adhesion chip (adapted from ref. 106). (D) Schematic diagram of the µCGA for 3D culture and single-cell drug screening (adapted from ref. 107). (E) The microfluidic cell trap array for single-cell anticancer drug screening (adapted from ref. 108).

Recent exciting advances in this field include the development of a high-throughput microdevice with 4000 ultraminiaturized wells to perform real-time, 3D cell invasion assays at different cell concentration gradients (Fig. 10A).^103^ In this platform, single cells or cells at various densities were trapped at the bottom of the wells by collagen gel, while FBS-loaded collagen gel as a chemoattractant was deposited on the top layer. Additionally, the microchip could be extended to obtain opposing gradients of two different cell types at the same time, as a controlled system to study the correlation between immune cell number and cancer cell invasion. This high-throughput microdevice can also be applied to efficiently screen candidate drugs that inhibit migration. To establish the microenvironment and chemotactic gradients, Agrawal's group developed a simplified strategy to manipulate the wettability of the desired interconnected channels, allowing introduction of extracellular matrix to mimic *in vivo* systems for real-time monitoring of individual migrating cells.^104^ They applied this novel on-chip platform to collectively investigate CTCs, including endothelial adhesion, extravasation, and migration of MDA-MB-231 breast cancer cells, through a 3D structure under a gradient of chemoattractant. Moreover, Qin's group designed a biomimetic microfluidic cytometry platform (Fig. 10B) that precisely separated cancer cells in a high-throughput manner. They further developed the term ‘transportability’ to describe the dynamic squeezing of cancer cells through micro-constrictions created by a matrix of trapping barriers comprising microgaps ranging from 15 µm to 4 µm in width.^105^ This guaranteed a promising microfluidic platform for profiling transportable phenotypes correlated with stiffness and cell-surface frictional force.^105^ This work emphasized the significance of biophysical properties, where cell transportability could be used as a potential marker to investigate cell heterogeneity and identify metastatic potential.

#### 4.4.2 Adhesion

Besides the investigation of single cell motility, Qin's group subsequently reported a high-throughput cell-adhesion chip with artificial microchannels coated with adhesive extracellular-matrix materials (Fig. 10C).^106^ According to their adhesive capability, cancer stem cell-like phenotypes with low adhesion were collected from the outlets while high-adhesive subtypes were trapped in the functionalized microdevice. The results suggested that cell adhesiveness could be utilized as a biophysical marker to effectively assess the stemness of CTCs.

#### 4.4.3 Drug response

In conventional drug response analysis, cells are treated with a new drug to test its preclinical safety and effectiveness. However, new drugs or treatment programs generally rely on the results of bulk analysis or large-scale clinical trials, which neglect individual variability, resulting in therapies that are only moderately effective, with the curative effects varying greatly from person to person. Therefore, high-throughput screening approaches for single CTCs would further advance individualized or personalized drug administration. Yang's laboratory developed a long-term single-cell analysis platform by manufacturing a high-throughput microcollagen gel array (µCGA), which not only provided 3D single-cell culture conditions, but also acted as an effective tool for revealing marked heterogeneity in drug response (Fig. 10D).^107^ Using the µCGA, the influence of proliferation dynamics for a series of drug concentrations was explored at the 3D single-cell level. Liu's group developed a microfluidic hydrodynamic trapping system based on bypass structures, achieving 90% efficiency for single-cell trapping (Fig. 10E).^108^ They then used a digital single-cell assay to assess the pharmacological efficacy of five commonly-used chemotherapeutic drugs. Furthermore, they gradually decreased the concentrations of the chemotherapeutic reagents over time to simulate physiological conditions, and evaluate whether cell apoptosis was promoted by the chemotherapeutic reagents *in vivo*.

Nevertheless, the above designs cannot screen different drug concentrations or different reagents on a single chip simultaneously, and thus they are not suitable for high-throughput or large-scale drug response analysis. To fill this gap, Liu's group subsequently fabricated a multifunctional gradient-customizing microfluidic chip for high-throughput single-cell multidrug resistance assays.^109^ The customized gradient profile was generated by varying the length of the distribution microchannels, instead of by serial dilution. The proposed single-step confluent gradient dilution device allowed investigation of drug cytotoxicity and chemo-sensitizing effects for as many as 64 different concentrations of drug. HepG2 cells were cultured at the single-cell level in a constant concentration of doxorubicin and a linear gradient of concentrations of cyclosporine A to investigate the chemo-sensitizing effects on doxorubicin sensitivity. This platform can offer multiple types of information on drug screening at single-cell resolution, including drug cytotoxicity, drug efflux kinetics and chemo-sensitizing effects. With the properties of lower dosage, good applicability and high throughput, this method forecasts a new promising horizon for high-throughput drug response analysis.

Despite the various advantages of the above-described phenotypic analyses, phenotype profiling is not yet ready for practical patient CTCs, presumably due to issues associated with the viability of released CTCs. A second challenge arises with the further exploration of relationships between biophysical characteristics and molecular mechanisms. The continued advancement of molecular and phenotypic profiling may help to gain a more profound and comprehensive understanding of metastasis.

## 5 Challenges and perspectives

CTCs are currently expected to become new biomarkers for cancer diagnostics and therapy assessment. However, single-cell analysis of CTCs is a relatively new and continually expanding area that may reveal multiple molecular observations as a basis for early cancer diagnosis and therapy monitoring in clinical trials. Nevertheless, due to the extremely low levels, heterogeneous biology and vulnerability of CTCs, there are still many factors affecting single CTC analysis results. For instance, choice of an isolation platform requires development of a method to achieve CTC enrichment with high throughput, purity, selectivity and viability. Immunocapture platforms usually provide higher enrichment purity, which could eliminate blood cell contamination. Yet, many of these methods may lose some of the CTCs with low expression of epithelial markers, resulting in the most invasive tumor cells with low or no epithelial marker expression being missed. The reality is more complicated, and some cancer cells may be in a partial mesenchymal state.^14^ In addition, marker-based strategies suffer from unsatisfactory throughput because of the long time required for sufficient binding between cells and capture ligands. Although physical-principle-based separation methods have the advantage of rapid analysis with high throughput, the heterogeneous biology of CTCs is still an inevitable issue. For example, the physical properties of CTCs may overlap those of blood cells, leading to loss of metastatic tumor cells accompanied by a higher proportion of blood cell contamination. Moreover, the side effects of physical-principle-based separations on cell viability caused by stress pressure, an electric field or conductive media must also be taken into consideration. Besides, some CTCs circulate as clusters, and their contribution to metastasis is still under investigation.^11^ Considerable genomic and proteomic heterogeneity does exist in CTC clusters as well as single CTCs.^110^ Another major challenge for CTC cluster research is that most existing isolation technologies are designed for capturing single cells rather than clustered CTCs. Therefore, platforms that integrate multiple capture principles may provide great utility, where their respective superiorities are combined and the major challenges of each platform may be avoided. Therefore, the above factors must be taken into consideration for developing methods to achieve complete CTC enrichment with high throughput, efficiency, purity, and viability for clinical diagnosis.

Single-cell analysis is still in need of significant further development in order to offer a comprehensive view of any single CTC. Single-cell analysis depends heavily on the quality of the isolated cells and will ultimately determine the appropriateness of the enrichment method. For example, in culture experiments and drug efficacy tests at the single CTC level, viability is the most significant factor. Thus, the separation process must maintain maximum cell viability. For genome and protein profiling, the purity of the CTCs is particularly important because of the need for high purity templates with minimal blood cell contamination to provide good materials for characterizing the heterogeneity of CTCs *via* large amounts of single-cell data. If the enrichment purity is not satisfactory, it will invariably introduce mysteries that complicate single cell data analysis.

Besides the challenges posed by CTC enrichment, the bottleneck of single-cell analysis technology also needs to be taken into consideration. The initial step in obtaining single-cell CTC characterization is the isolation of individual cells from a captured population. While the methods for isolating single cells from the rare released or captured cells (about 10 to 10^3^) remain a formidable technical challenge, several available isolation methods have been employed: flow-activated cell sorting (FACS), micromanipulation, optical tweezers and microfluidics isolation. Compared with other methods, microfluidics isolation, which is currently very popular, has several attractive features, such as high throughput, low sample and reagent consumption, and more importantly, the integration of enrichment and analysis systems. Microfluidic approaches enable individual cells to be isolated into small volumes varying from a few tens to a few hundreds of picoliters, thus providing several benefits, including short molecular diffusion time, a relative increase in the concentration of cellular analyte, and the possibility to investigate many cells in parallel. However, single CTC isolation efficiency is the main issue. CTCs are present in very low concentrations in the blood, and are therefore extremely precious materials for analysis. Of course, a lower single cell isolation efficiency will have a correspondingly larger loss of valuable and rare CTCs, resulting in the unavailability of comprehensive information about the captured CTCs.

Another important direction in this area includes simultaneous multiplexed profiling and throughput increase in order to achieve multiplexed analysis of thousands of CTCs in parallel with substantially lower cost. A recent developing technology uses cellular barcoding techniques to profile 10 000–100 000 single cells in parallel in order to reduce cost and increase throughput.^111^ While most single-cell sequence analyses are still cost prohibitive for ordinary patients, scientists are looking forward to largely overcoming this barrier in the next few years, as the cost of next-generation sequencing technology is showing a remarkably steady decline due to new technical innovations and intense industrial competition.

As development and breakthroughs occur, the realization of integrated microfluidic devices is expected in the near future. These will allow efficient CTC enrichment and single-CTC profiling to ensure that genomic and proteomic information at the single-cell level reflects the true characteristics of captured CTCs, and that no artificial operation is introduced during sample handling. Such dreams can be realized through the efforts of researchers in chemistry, proteomics, genomics, biology, bioinformatics, bioengineering, and medicine working together and will have a significant impact on cancer cell biology and precision medicine.
